# Cold weather increases the risk of scrotal torsion events: results of an ecological study of acute scrotal pain in Scotland over 25 years

**DOI:** 10.1038/s41598-020-74878-0

**Published:** 2020-10-21

**Authors:** Chidi N. Molokwu, Judith K. Ndoumbe, Chris M. Goodman

**Affiliations:** 1 Urology Department, Ninewells Hospital, Dundee, DD1 9SY UK; 2 Present Address: Urology Department, Bradford Teaching Hospitals, Bradford, BD9 6RJ UK; 3 Valleyfield Health Centre, Dunfermline, KY12 8SJ UK

**Keywords:** Ecophysiology, Testis, Urological manifestations, Urology, Epidemiology

## Abstract

The role of ambient temperature in the aetiology of acute scrotal pain (ASP) remains uncertain. The most common causes of ASP are torsion of the testis (TT) or its appendages (TA) and epidymo-orchitis (EO). We undertook an ecological study of ASP in Scotland to determine whether a seasonal variation could be observed. Episode reports for TT, TA and EO in Scotland over 25 years were collated monthly. Statistical analyses were performed to determine whether changes in ambient temperature during the year could explain variations in monthly frequency. 7882 episodes of TT and TA (Group A), and 25,973 episodes of EO (Group B) were reported. There was significant variance in the frequency of Group A (*p* < 0.0001) and B (*p* = 0.0031) episodes by month, higher frequency of Group A episodes in the colder half of the year (*p* < 0.0001), and an inverse correlation between the frequency of Group A episodes and ambient temperature (Spearman r = − 0.8757, 95% CI − 0.9661 to − 0.5941, *p* = 0.0004). Ambient temperature is likely to be playing a role in the aetiology of TT and TA in Scotland but not EO. Further study is warranted to explain underlying mechanisms.

## Introduction

Acute scrotal pain (ASP) is a medical emergency. The most common causes of ASP are testicular torsion (TT), torsion of the testicular appendages (TA), and epididymo-orchitis (EO)^1^. Acute scrotal pain requires urgent medical assessment and surgical exploration of the scrotum if testicular torsion cannot be ruled out clinically. TA can be difficult to diagnose without exploration, leading to a high rate of diagnosis at exploration^2^. EO is managed with appropriate antimicrobials, determined by the clinical history and results of microbiological culture.

Seasonal variations have been reported for several medical conditions, with a number of pathophysiological processes explaining such findings. Reports on the seasonality of ASP and TT have been contradictory, with some studies showing evidence of seasonality and other studies not^3^. It has been shown that decreasing temperature causes increased contraction of the cremasteric muscles^4,5^, and this may lead to an increase in the frequency of TT in winter months^3^. We performed a large ecological study of ASP in Scotland, which lies within the temperate latitudes, to determine if seasonality of presentation could be identified.

## Results

**Figure 1:**
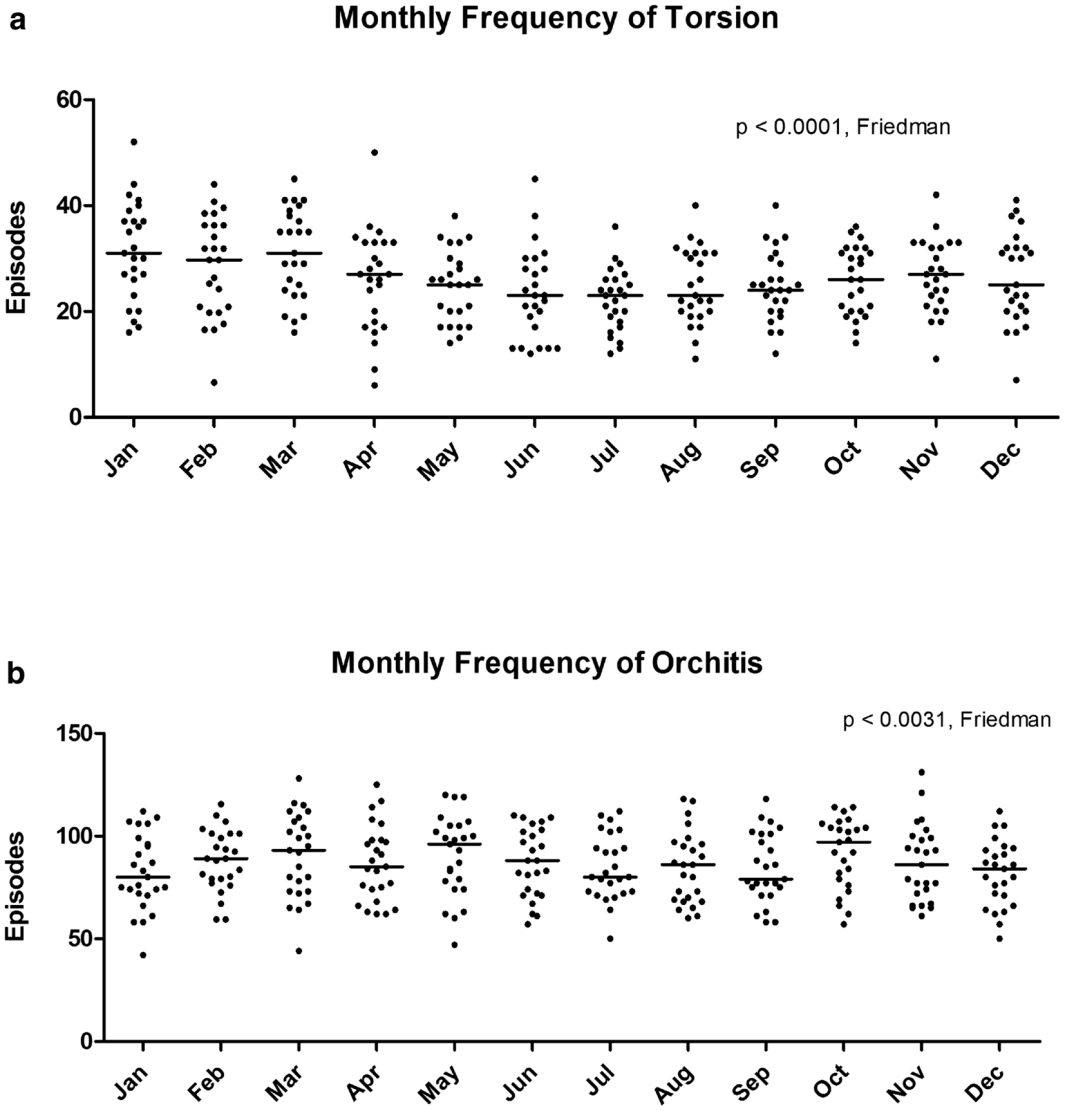
Monthly frequency of (**a**) Torsion (TT and TA) and (**b**) Orchitis (EO) episodes over the 25 year period. Each data point represents the number of episodes recorded per month equalised to a 30-day month. Bars represent median values.

**Figure 2:**
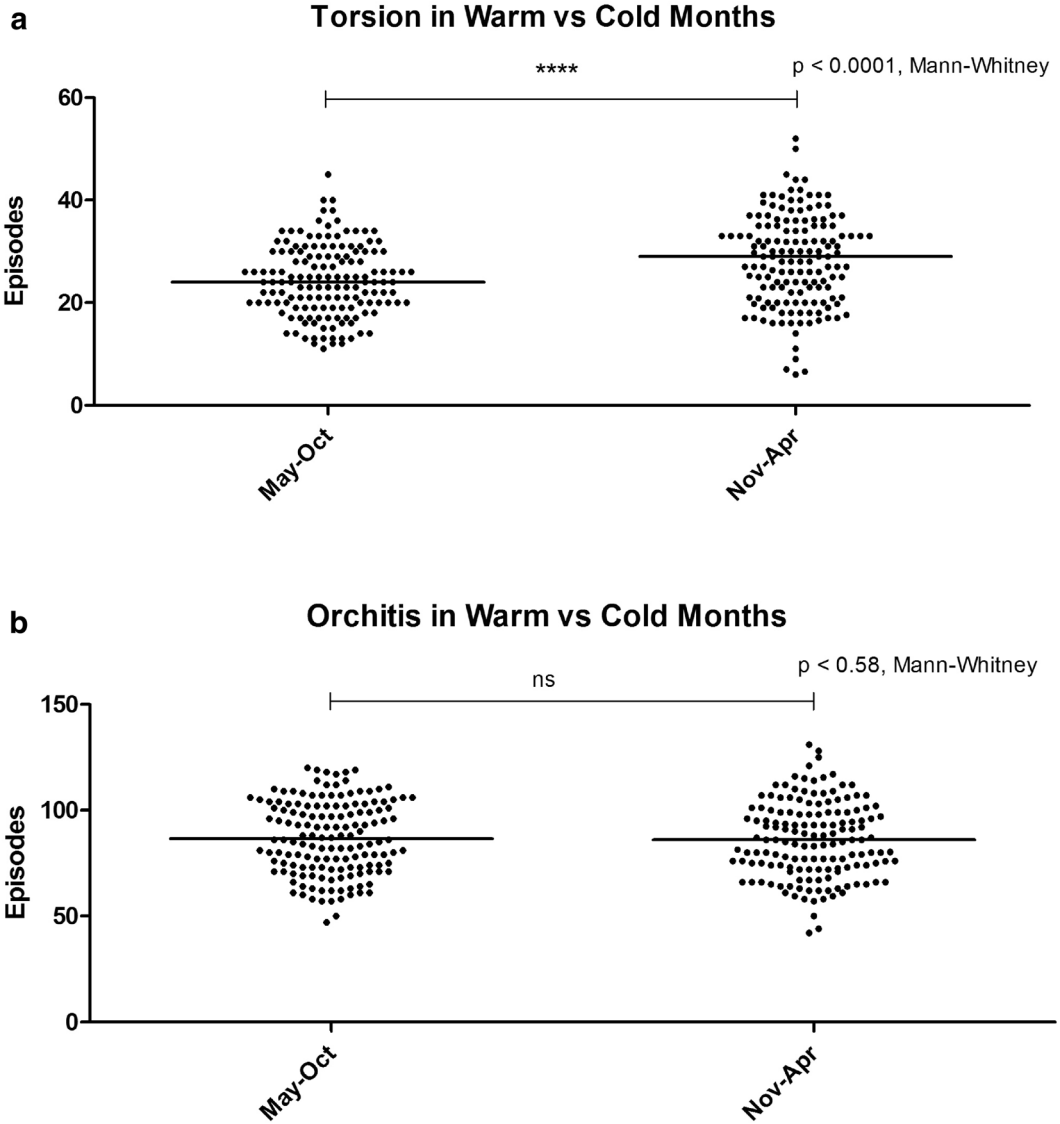
Comparison of the frequency of (**a**) Torsion (TT and TA) and (**b**) Orchitis (EO) during the warmer half (May to October) versus the colder half (November to April) of the year. Each data point represents the number of episodes recorded equalised to a 30-day month and the bars represent the median values.

**Figure 3:**
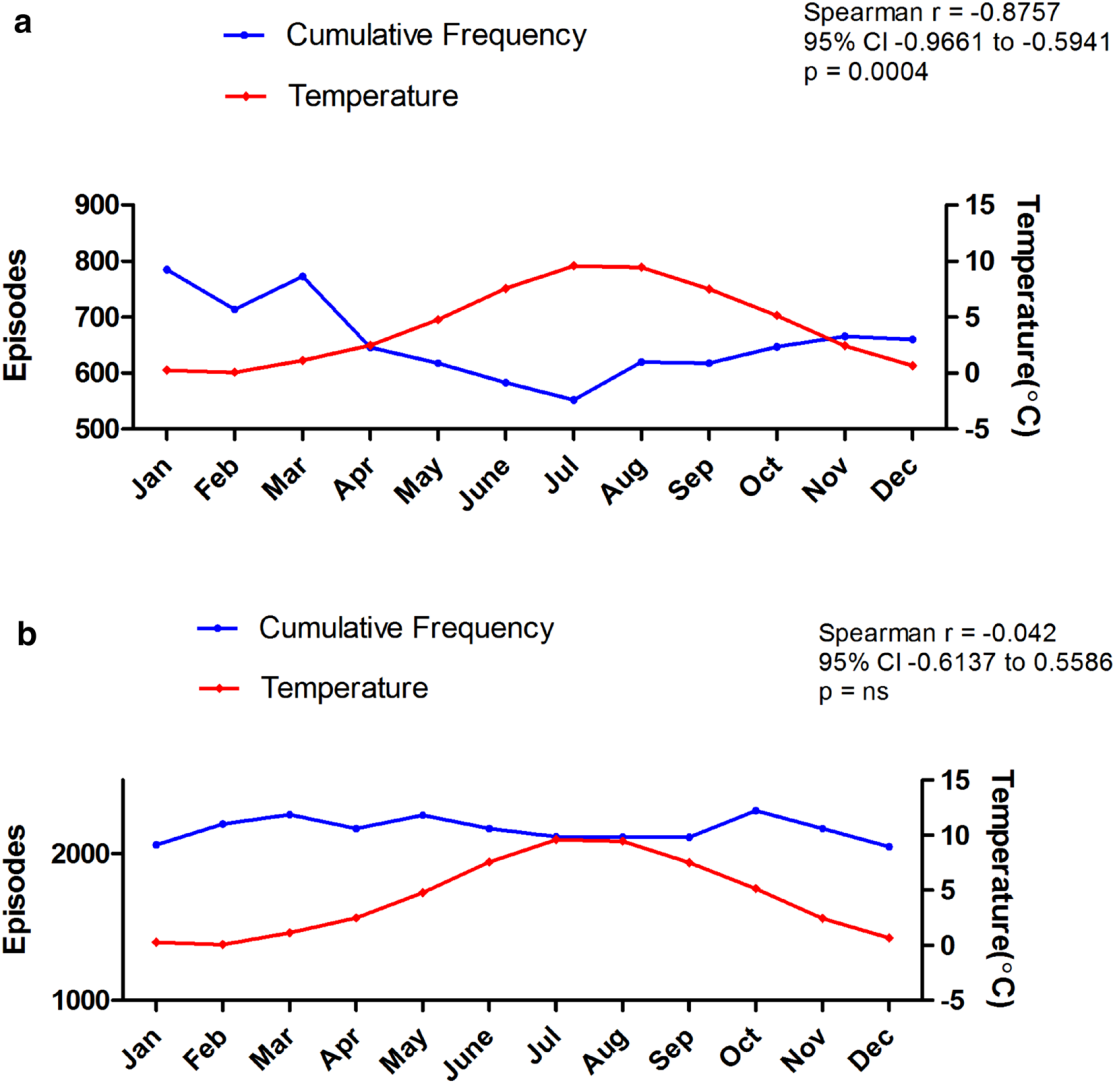
Correlation of the cumulative monthly frequency of (**a**) Torsion (TT and TA) and (**b**) Orchitis (EO) with the average ambient temperature over the 25 years. The frequency for each month was equalised to a 30-day month.

In the 25 year period from 1983 to 2007, there were 33,855 episodes coded as N44 and N45 identified from ISD records. Of these 7882 (23%) were TT and TA (Group A), and 25,973 (77%) were EO (Group B). May to October were the warmest (range 4.8–9.6 °C) and November to April the coldest months (range 0.1–2.5 °C). We found a significant variance in the frequency of Group A episodes by month (*p* < 0.0001) with higher frequency in the colder months (Fig. 1a), and Group B episodes (*p* = 0.0031) with highest frequency in March, May and October (Fig. 1b). There was a significantly higher frequency of Group A episodes in the colder months compared to the warmer months (*p* < 0.0001), but no difference for Group B episodes (*p* = 0.58) (Fig. 2a, b). We also found that there was a significant inverse correlation between the frequency of Group A episodes and ambient temperature (Spearman r = − 0.8757, 95% CI − 0.9661 to − 0.5941, *p* = 0.0004), but no correlation with ambient temperature for Group B episodes (Spearman r = − 0.04203, 95% CI − 0.6173 to 0.5886, *p* = 0.9039) (Fig. 3a, b).

## Discussion

In this study we analysed data from monthly ASP episodes over a 25 year period to determine if there was a seasonal pattern of presentation. We found a seasonal variation in scrotal torsion events (TT and TA), with higher frequency in the colder months, and an inverse correlation between monthly frequency and ambient temperature. There was increased frequency of EO in March, May and October but no correlation with temperature.

There has been interest in the seasonality of TT for many years^6^. Decreasing temperature causes increased contractility of the cremasteric muscles^4,5^, which may lead to an increase in the frequency of TT in colder months. Previous studies have yielded conflicting results^3^. Most of these studies reported case series of a relatively small number of patients (n = 39 to n = 2876). A large study from Brazil^3^ analysing 21,289 episodes of TT found seasonality of presentation with higher incidence in colder months, which was more significant in the more temperate regions than tropical regions of Brazil. A previous report from Dundee in Scotland showed an increased frequency of TT during the colder months from a series of 173 patients^1^. The present report is a larger study involving 33,855 episodes, of which 7882 had torsion events, and provides more robust evidence of seasonality of torsion.

Seasonal variation in the frequency of EO has not previously been reported to our knowledge. We were unable to explain the increased frequency of EO in March, May and October within our dataset. Further epidemiological study will be required to elucidate the reasons. Possibilities to consider include sexual behaviour patterns of the male population.

Limitations of this study include the use of data from a large public database with well reported advantages and disadvantages^7^, and the ecological fallacy, meaning that it may not be appropriate to apply these generalised population-based findings to individual patient care.

We do not suggest, based on our findings, that the threshold for surgical exploration be raised for patients with ASP presenting during warmer months. Public health measures could be considered, for example encouraging the wearing of warm clothing and undergarments by young males during colder months may reduce the frequency of TT and TA, as the style of clothing could have a direct effect on scrotal temperature^8^.

In conclusion, the findings of this large ecological study provide further robust evidence of seasonality of ASP, with the frequency of torsion events correlating negatively with ambient temperature. Further study is required to explain monthly variations in presentation of EO.

## Methods

### Data sources

All episode reports for the three most common causes of ASP namely torsion of the testis (TT), torsion of appendages (TA) and epididymo-orchitis (EO) in Scotland for the 25 year period from 1983 to 2007 was obtained from the Information Services Division (ISD) of the National Health Service (NHS), Scotland. There are currently 14 Health Boards in Scotland, covering well defined geographical areas with 2008 population figures ranging from 9,789 in Orkney to 573,021 in Greater Glasgow and Clyde^9^ (Table 1). Each Health Board collects episode data from all hospital and community practice attendances and these are coded and collated centrally by ISD. All intra-scrotal torsion events (including TT and TA) were coded under N44, while EO was coded as N45 using the International Classification of Disease version 9 (ICD-9). Management of N44 episodes usually require surgical exploration, therefore they were left in one group (Group A). EO is usually managed non-surgically and was analysed as a separate group (Group B). The monthly frequency of presentation from each Health Board was collated for all 25 years. Data for each month was equalised to a 30-day month. Ambient temperature data for the period was obtained from the United Kingdom Meteorological Office (Met Office).

**Table 1:** The health boards in Scotland showing the population of each health board for 1998, and 2008.

NHS health board	Total population 1998	Total population 2008	Male population 2008
Ayrshire and Arran	371,790	367,510	175,832
Borders	106,040	112,430	54,451
Dumfries and Galloway	148,740	148,580	71,926
Fife	346,540	361,815	174,608
Forth valley	276,970	290,047	140,043
Grampian	528,670	539,630	268,196
Greater Glasgow and Clyde	1,210,820	1,194,675	573,021
Highland	300,520	309,900	152,693
Lanarkshire	555,180	561,174	269,818
Lothian	767,920	817,727	394,510
Orkney	19,590	19,890	9,789
Shetland	22,700	21,980	11,099
Tayside	394,050	396,942	191,338
Western Isles	27,540	26,200	12,881
Total	5,077,070	5,168,500	2,500,205

### Statistical analysis

Statistical analyses were performed to determine whether there was a seasonal variation in the presentation of Group A and Group B events, and whether differences in ambient temperature during the year could explain any variation observed. The Friedman test was used to analyse for variance in the monthly frequency of episodes. The Mann–Whitney test was used to compare the frequency of episodes between the colder and warmer months, and the Spearman test was used to determine if there was a correlation between the cumulative monthly frequency of episodes and average monthly ambient temperature. All statistical analyses were performed using GraphPad Prism 5 software (GraphPad Software Inc., San Diego, CA, USA).
